# Oxidative Status and Lipofuscin Accumulation in Urothelial Cells of Bladder in Aging Mice

**DOI:** 10.1371/journal.pone.0059638

**Published:** 2013-03-20

**Authors:** Martina Perše, Rade Injac, Andreja Erman

**Affiliations:** 1 Medical Experimental Centre, Institute of Pathology, Faculty of Medicine, University of Ljubljana, Ljubljana, Slovenia; 2 Institute of Pharmaceutical Biology, Faculty of Pharmacy, University of Ljubljana, Ljubljana, Slovenia; 3 Institute of Cell Biology, Faculty of Medicine, University of Ljubljana, Ljubljana, Slovenia; Leibniz Institute for Age Research - Fritz Lipmann Institute (FLI), Germany

## Abstract

Age-related changes in various tissues have been associated with the onset of a number of age-related diseases, including inflammation and cancer. Bladder cancer, for instance, is a disease that mainly afflicts middle-aged or elderly people and is mostly of urothelial origin. Although research on age-related changes of long-lived post-mitotic cells such as neurons is rapidly progressing, nothing is known about age-related changes in the urothelium of the urinary bladder, despite all the evidence confirming the important role of oxidative stress in urinary bladder pathology. The purpose of this study was thus to investigate the oxidative status and age-related changes in urothelial cells of the urinary bladder of young (2 months) and aging (20 months) mice by means of various methods. Our results demonstrated that healthy young urothelium possesses a powerful antioxidant defence system that functions as a strong defence barrier against reactive species. In contrast, urothelial cells of aging bladder show significantly decreased total antioxidant capacity and significantly increased levels of lipid peroxides (MDA) and iNOS, markers of oxidative stress. Our study demonstrates for the first time that ultrastructural alterations in mitochondria and accumulation of lipofuscin, known to be one of the aging pigments, can clearly be found in superficial urothelial cells of the urinary bladder in aging mice. Since the presence of lipofuscin in the urothelium has not yet been reported, we applied various methods to confirm our finding. Our results reveal changes in the oxidative status and structural alterations to superficial urothelial cells similar to those of other long-lived post-mitotic cells.

## Introduction

The urothelium of the urinary bladder is a three layered epithelium covering the luminal side of the urinary bladder. The urothelium is one of the slowest cycling epithelia in the body. Murine urothelium is known to proliferate extremely slowly in normal adult animals [1]–[3], which makes studying its kinetics *in vivo* difficult and many questions concerning the proliferative organization and proliferation activity in the urothelium are thus still unanswered. Labelling indices are known to vary between 0.5% and 1%, cell cycle time is about one year and mitotic cells are mostly present in deeper layers and never in the superficial layer [1]–[3]. The urothelium contains a low proportion of cycling cells capable of resuming cell cycle progression when required. It also contains a low proportion of post-mitotic superficial cells, which are incapable of further proliferation. It has been demonstrated that terminally differentiated superficial cells in murine urinary bladder have a lifespan of more than 200 days [2], [4], which suggests that superficial cells may be subject to age-related changes responsible for various urinary bladder disorders.

Accumulated evidence suggests that aging in long-lived post-mitotic cells, such as neurons, is associated with increased production of nitrogen or oxygen reactive species, resulting in increased products of lipid peroxidation, such as malondialdehyde (MDA), and increased oxidation of proteins and genetic material [5]. Oxidative conditions cause progressive structural and functional alterations of cellular organelles, such as mitochondria and lysosomes, and changes in redox-sensitive signalling processes [6]–[8]. These harmful conditions contribute to increased susceptibility to a variety of diseases, including inflammation and cancer [9]. Oxidative stress as a consequence of increased production of nitrogen or oxygen reactive species has also been demonstrated in bladder tumour cells, urinary tract infection and ischemia/reperfusion injury in the bladder [10]–[13].

To counteract the detrimental effects of increased formation of reactive species, cells possess a powerful and complex antioxidant defence system composed of numerous endogenous antioxidants. When the production of reactive species increases, cells in turn stimulate antioxidants to prevent cell damage [7]. Decreased levels of antioxidants have been reported in urinary bladder cancer, urolithiasis and sepsis [11], [13], [14]. It has been suggested that lipid peroxidation plays an important role in the development of urolithiasis [15].

It has also been suggested that the antioxidant defence system weakens with age, which significantly contributes to the above mentioned oxidative conditions in aged cells [5]. Age-related changes in different tissues have been associated with the onset of various age-related diseases [5], [7]. Although research into and knowledge about age-related changes in post-mitotic cells, such as neurons [16] or vascular endothelial cells [17], is rapidly progressing, nothing is known about age-related changes in urothelial cells of the urinary bladder, despite all the evidence confirming the important role of oxidative stress in urinary bladder pathology. Age-related diseases of the urinary bladder, such as bladder cancer or cystitis, are frequent disorders in humans, causing significant clinical and financial problems for the health system. Bladder cancer, for instance, is a disease that mainly afflicts middle-aged or elderly people and is mostly of urothelial origin, termed urothelial carcinoma [18].

In view of all the aforementioned, an insight into age-related changes in this tissue is of great interest. To our knowledge, no research on age-related changes in the urothelium of the bladder has been performed. We believe that such information may be crucial for understanding the initiation of pathological processes in the urinary bladder.

The purpose of this study was thus to investigate oxidative status and age-related changes in urothelial cells of the urinary bladder of young (2 months) and aging (20 months) mice. Differences in the oxidative status in urothelial cells were evaluated by measuring the activities of the antioxidant defence system, such as superoxide dismutase (SOD), catalase (CAT), glutathione peroxidase (GPx), glutathione reductase (GR), glutathione (GSH), glutathione disulphide (GSSG) and total antioxidant status (TAS) and by the levels of markers of oxidative changes, such as MDA, inducible nitric oxide synthase (iNOS) and cytochrome c oxidase (COX). We also investigated differences in apoptosis, proliferation and differentiation and structural alterations of cell organelles in urothelial cells of the bladder in young and aging mice.

We demonstrated for the first time that superficial urothelial cells of aging bladder show changes in oxidative status and structural alterations that accumulate over time, similar to those of other long-lived post-mitotic cells in the aging process.

## Results and Discussion

Since we found no data in the literature regarding levels of antioxidants in mouse urothelium, we made preliminary measurements to evaluate whether the antioxidant status in mouse urothelium can be determined quantitatively, despite the very small tissue sample, i.e., only a few micrograms of tissue per mouse, since a previous investigation of antioxidants in pig urothelium raised some questions about the test approach [19]. We made some modifications to our previously reported method [20], including manual homogenization (strongly recommended) and a longer homogenization time (ca. 10 to 15 minutes) without dilution of the homogenized samples, which resulted in very small amount of test solution (2–3 µl) and this in turn influenced the amount of substrate/reagents used for sample preparation.

We found no data in the literature about testing antioxidants in the urothelium of mouse bladder, which of course raised many questions about the test approach. The major concerns at the beginning of our preliminary investigation were the small tissue sample size and the analytical sensitivity of the method used. Very careful preliminary investigation was performed based on our previous knowledge and experience from testing lung tissues, since lung tissue is not rich with cells and the level of oxidative parameters is very low. After the homogenization step, we avoided dilution of the samples, which resulted in very low amount of solution for testing (2–3 µl). Consequently, a lower amount of substrates was used in these analyses. For example, if the sample size according to the kit was supposed to be 1 ml and the total reagent volume 10 ml, we reduced it to 3 µl and 300 µl, respectively. In the case of CAT, the amount of reagent was not even proportionally reduced because the calculation for enzyme activity also took into account the volumes of sample and reagent used. In order to achieve an acceptable linearity in the CAT case, therefore, especially for the urothelium of a young mouse, the reagent amount used was lower so as to achieve a small diluted rate of CAT in the analysed mixture. For example: instead of 1 ml of sample and 10 ml of reagent, we used 3 µl of sample and 250 µl of reagent, rather than a 3 µl and 300 µl approach. Such preparation allowed testing within acceptable linearity. We had no problems with other parameters in terms of results and limits. The easiest to test were MDA and GSH, since the very sensitive detection capacity of the HPLC made it more than suitable. The results were far from the LOD (limit of detection) and LOQ (limit of quantification). In the case of GSH, the amount of internal standard was reduced from 150 µl to 30 µl, so as to have less difference between the area of peaks (AUC; area under the curve) and high differences among samples, standard and internal standard, as well as error, for the sake of program integration of the results and areas.

The relative standard deviation (RSD or CV%) was calculated to show intra-subject variability as well as the method assay accuracy. According to international requirements for *in vivo* testing [21], all parameters (drugs, blood markers etc.) are acceptable within the range of CV% below 30%. Within the group variability (young and old mice) for all investigated parameters, the CV% was between 2 and 23%, which showed acceptable *in vivo* variability and also the suitability of the selected group size (n = 12 for tissue testing and n = 6 for serum testing) in our study.

On the other hand, a CV% below or equal to 2% [22] confirms the accuracy of assay by ICH and a CV% below or equal to 5% confirms the robustness of testing (different equipment, analysts, reagents, analytical columns and plates, mobile phase etc…) [23]. For highly variable methods, this can even be up to 15%.

Very sensitive and accurate HPLC methods for MDA and GSH determination were used in our study, while all other parameters were tested using a more variable spectrophotometric method. In the optimisation phase, 2, 3, and 5 repetitions were used for HPLC testing and the CV% was below 1.45% in all cases. In view of that, we used only two repetitions in the main study. The same approach was used for the spectrophotometric method (TP, SOD, CAT, GR, GPx, TAS, LDH) and the CV% was between 5 and 10% in all cases, no matter how many repetitions (2–5). In view of that, a two repetition approach was used. The high analytical variability of the spectrophotometric results is connected to the very small sample sizes (a few microliters) as well as the non-robust methodology. Altogether, the analytical and group size approach was found to be suitable and within the acceptable range of CV%.

Linearity between the tissue used and analytical outcome for each parameter cannot be shown, since the behaviour differs within each group, as well as for each of the parameters. Optimization before any official testing is strongly recommended.

After the aforementioned modifications, the levels of antioxidants in the urothelium of mouse bladder were sufficiently high to measure successfully all the mentioned parameters within the linearity range, as well as above the limit of quantification. Although this approach allowed us to make two repetitions for each parameter, the small amount of mouse urothelium only enabled us to analyse a limited number of antioxidants (i.e., all the parameters analysed in our study).

### Oxidative Status

The antioxidant defence system is composed of numerous antioxidants, which work collectively. Antioxidants are divided into primary (SOD, CAT, GPx, GR), secondary (vitamin E, vitamin C, beta-carotene, uric acid, bilirubin, and albumin) and tertiary (biomolecules damaged by free radicals) defence elements in the cell [7]. In order to evaluate the total antioxidant capacity in the urothelium of young and aging mice, we analysed the parameters of antioxidant defence (i.e., SOD, CAT, GPx, GR, GSH, GSSG), as well as levels of TAS, an important diagnostic guide [24], [25]. All measured parameters were significantly elevated in the urothelium of aging mice (Fig. S1), demonstrating that the total antioxidant capacity in the urothelium of aging mice was impaired (Fig. S2). Additional study showed that the differences in the activity of some antioxidant enzymes can already be found in the urothelium of 12-month old mice (Table S1). In contrast to the urothelium, no differences in the activity of the antioxidant defence system in the serum were found between young and aging mice (Table 1), suggesting that the environment in aging urothelium may be modified by oxidative changes [6], [26].

**Table 1:** Age-related differences in the levels of antioxidants, lactate dehydrogenase and malondialdehyde in the urothelium and serum of young and aging C57BL/6JOlaHsd female mice.

	Urothelium			Serum		
Parameter	Young(n = 12)	Aging(n = 12)	P value	young(n = 6)	aging(n = 6)	P value
**TP (g/L)**	6.2±1.2	8.6±0.9	ns	72.2±5.3	69.4±5.2	ns
**SOD (U/mg TP)**	7.13±1.32	8.15±1.38	ns	0.33±0.01	0.29±0.02	ns
**CAT (U/mg TP)**	0.16±0.01	1.08±0.14	<0.001	0.31±0.07	0.06±0.01	0.006
**GR (U/g TP)**	1.64±0.35	5.15±0.32	<0.001	0.26±0.06	0.24±0.05	ns
**GPx (U/g TP)**	10.8±1.9	29.0±2.8	<0.001	10.9±0.7	11.8±0.6	ns
**free GSH (µmol/L)**	237.8±6.4	516.9±19.5	<0.001	264.7±8.9	270.6±22.4	ns
**Total GSH (µmol/L)**	1364.4±31.1	2743.0±118.6	<0.001	1317.2±48.6	1295.1±34.5	ns
**GSSG (µmol/L)**	563.3±15.9	1113.1±55.0	<0.001	526.2±24.9	512.3±19.4	ns
**GSH/GSSG**	0.43±0.02	0.48±0.03	ns	0.51±0.04	0.54±0.06	ns
**TAS (mM)**	24.7±0.9	10.3±0.5	<0.001	n.a.	n.a.	
**LDH (U/L)**	381.3±51.4	1009.3±110.1	<0.001	130.6±9.5	256.7±54.2	0.010
**MDA (µg/L)**	210.8±9.6	607.1±27.3	<0.001	32.6±1.7	22.6±2.9	0.016

Significant differences between groups of young and aging mice were evaluated by a test of independence (t test) and when variances of the two samples were different, we used the Mann-Whitney (Wilcoxon) W test. The results were considered statistically significant at P<0.05.

Values are mean ± SEM.

Legend: n.a., not analysed; ns, non-significant; CAT, catalase; GPx, glutathione peroxidase; GR, glutathione reductase; GSH, glutathione; GSSG, glutathione disulphide; LDH, lactate dehydrogenase; MDA, malondialdehyde; SOD, superoxide dismutase; TAS, total antioxidant status; TP, total protein concentration.

We therefore analysed MDA, a commonly used marker of oxidative alterations of lipids and one of the first signs of oxidative stress, which is a common age-related feature found in neurons [5] and myocites [27]. Analysis revealed that aging mice had no changes in the serum levels of MDA but significantly higher urothelial levels of MDA (almost 3-fold) than young mice (Table 1), demonstrating age-related and tissue-specific differences in oxidative status. Lipid peroxidation reflects oxidative tissue damage, which results in structural alteration of membranes and functional impairment of cellular structures [26], [28] and has been reported as a pathogenic factor in a variety of diseases [26].

**Figure 1:**
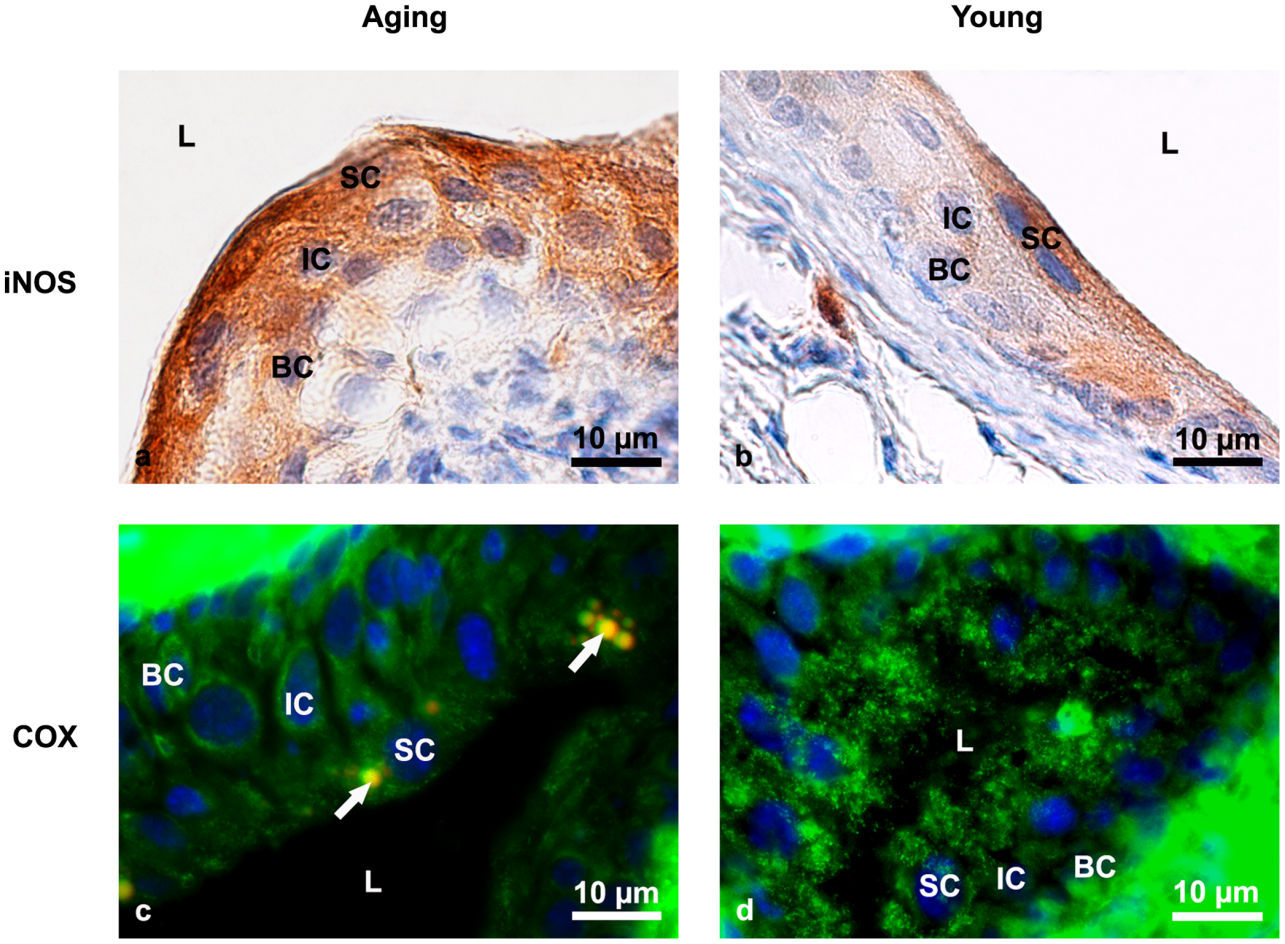
Immunolabeling of iNOS and COX in aging and young mice urothelium. (**a**) The intense brown colour shows a strong positive immunoreaction against iNOS in the cytoplasm of superficial, intermediate and basal urothelial cells of aging mice. Nuclei are stained with haematoxylin (blue). (**b**) Only occasional iNOS-positive superficial cells with pale brown-coloured cytoplasm are detected in the urothelium of young mice, while intermediate and basal cells are almost completely iNOS-negative. Nuclei are stained with haematoxylin (blue). (**c**) Weak immunoreaction (green fluorescence) against COX in all urothelial cells of aging mice. Nuclei are stained with DAPI (blue). Arrows show lipofuscin granules. (**d**) Intense and extensive dotty reaction (green fluorescence) against COX in the cytoplasm of superficial urothelial cells of young mice. Nuclei are stained with DAPI (blue). Arrows show lipofuscin granules. SC-superficial cells, IC-intermediate cells, BC-basal cells, L-lumen of the urinary bladder.

We further evaluated the induction of iNOS, which can be used as an indication of an increased oxidative environment [29]. Immunolabelling against iNOS revealed a strong reaction in the cytoplasm of all urothelial cells of aging mice, with the strongest reaction in the cytoplasm of superficial cells, indicating that oxidative changes were the most intensive in superficial cells. In contrast, iNOS immunoreaction in the urothelial cells of young animals was negative, with the exception of a few individual weak positive superficial cells (Fig. 1a, b). Increased levels of iNOS have already been found associated with bladder inflammation and cancer [10], [30]. In an oxidative environment, proteins are a frequent target of oxidative alterations, which may result in decreased protein turnover. The latter is one of the proposed underlying mechanisms of aging [31]. We therefore performed immunolabelling against mitochondrial COX, the terminal enzyme complex of the electron transport chain in the inner mitochondrial membrane, which showed a weak and less comprehensive positive reaction in superficial cells of aging mice in comparison to the strong reaction in the entire cytoplasm of superficial urothelial cells of young mice. No such difference was found in the intermediate and basal urothelial cells of aging or young urothelium (Fig. 1c, d). Decreased staining intensity of COX in aging urothelium suggests a reduced content of this enzyme in mitochondria. Based on the increased activity of both hydrogen peroxide-removing enzymes (i.e., GPx (3-fold) and particularly CAT (6-fold)), it is possible that COX was subjected to oxidative damage as a result of oxidative modifications in mitochondria. In addition, the depletion of COX content may also suggest a decreased mitochondrial content or mitochondrial dysfunction [32]. It is well established that ROS can increase as a consequence of normal aging processes, by which mitochondria lose efficiency because of age-dependent changes [8].

Nevertheless, gradual oxidative damage of macromolecules (lipids, proteins) clearly indicates that the antioxidant defence was unable to prevent oxidative damage in the urothelium. However, was this oxidative damage extensive enough to trigger apoptosis or necrosis of cells? It is well documented that, depending on the severity of oxidative stress, cells may undergo gradual structural alterations, or apoptosis or even necrosis may be induced [6]. We thus assumed that excessively high levels of oxidative stress would result in increased apoptotic activity or necrosis of superficial urothelial cells. This in turn would induce intense proliferative activity and differentiation of urothelial cells.

**Figure 2:**
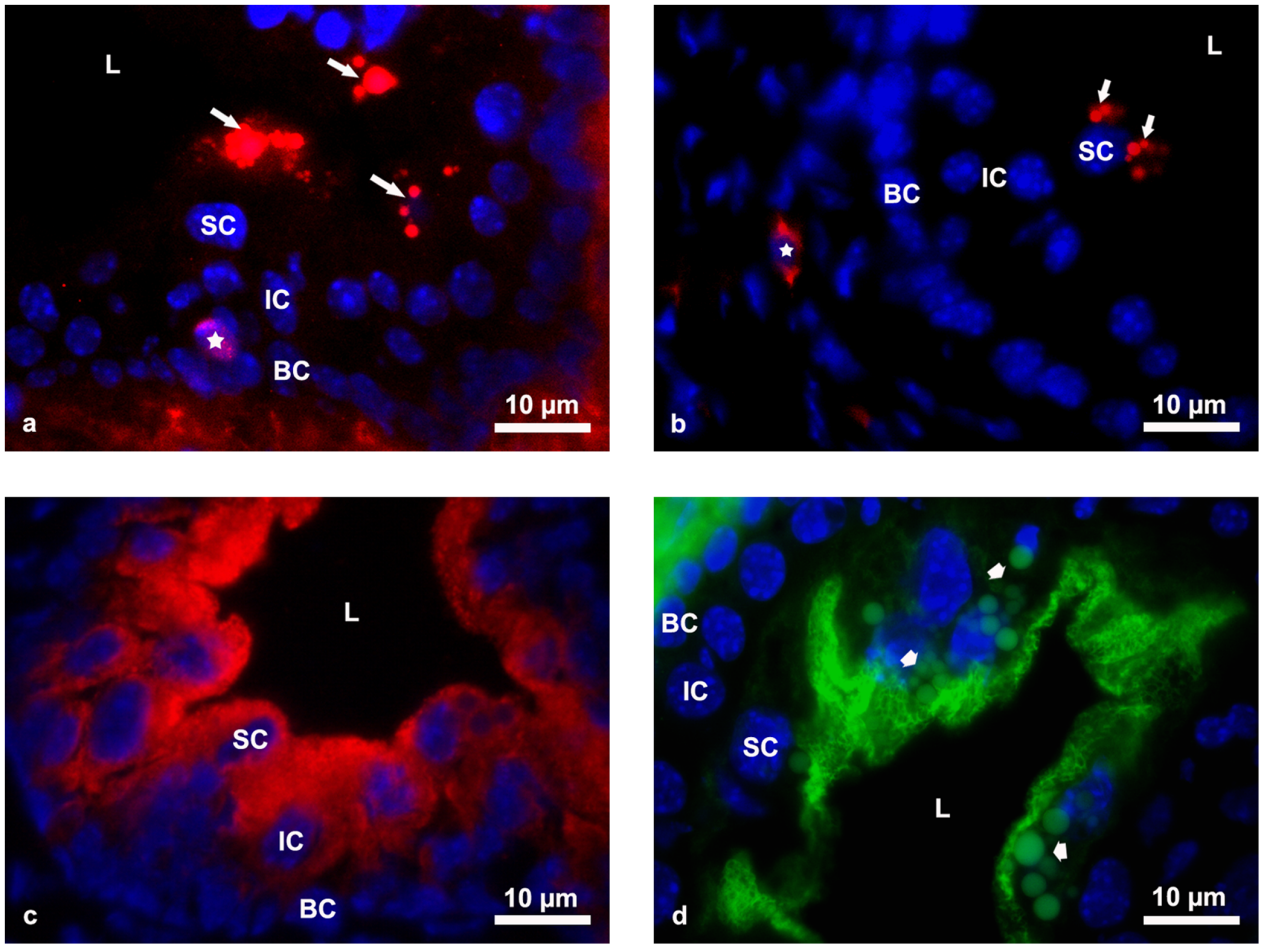
Immunolabeling of Ki-67, active caspase 3, uroplakins and cytokeratin 20 in aging mice urothelium. (**a**) Individual Ki-67 positive cells with red fluorescent signal (asterisk) are present in the urothelium of aging mice. Nuclei are stained with DAPI (blue). Arrows show lipofuscin granules. (**b**) No apoptotic cells (red fluorescence) were detected in the urothelium. Apoptotic fibroblast in connective tissue is marked with asterisk. Nuclei are stained with DAPI (blue). Arrows show lipofuscin granules. (**c**) Immunoreaction against uroplakins shows typical labeling (red fluorescence) of the apical plasma membrane and the membranes of specific vesicles - fusiform vesicles in the cytoplasm of superficial and intermediate cells. Nuclei are stained with DAPI (blue). (**d**) Immunoreaction against cytokeratin 20 (green fluorescence) reveals typical network of this cytokeratin in the apical cytoplasm of superficial cells. Nuclei are stained with DAPI (blue). Arrows show lipofuscin granules. SC-superficial cells, IC-intermediate cells, BC-basal cells, L-lumen of the urinary bladder.

In order to test our assumption, we evaluated potential changes in the proliferation, differentiation and apoptosis of urothelial cells by electron microscopy analysis and immunolabelling. The selection of immunostaining markers was based on previous results of our studies on the embryonic and postnatal development of the urothelium, with an emphasis on cell proliferation, differentiation and cell death [33]–[35]. Immunofluorescent labelling of Ki-67 and active caspase 3 and observations from transmission electron microscopy revealed no increase in proliferation activity or apoptotic activity of aging urothelium in comparison to the urothelium of young animals (Fig. 2a, b). Immunofluorescent detection of uroplakins and cytokeratin 20, as the main markers of superficial urothelial cell differentiation [36], showed the same supramolecular organization and localization of both markers in the urothelium of young and aging mice (Fig. 2c, d). These results suggest that the survival of urothelial cells in aging bladder was not compromised. This fits well with the increased activity of antioxidant enzymes (CAT, GPx, GR) and unchanged GSSG/GSH ratio found in aging urothelium. Changes in the GSH/GSSG ratio are frequently used as an indicator of oxidative stress [37]. On the other hand, a weakened antioxidant defence and increased markers of oxidative stress (increased MDA and iNOS) clearly demonstrate that the balance between pro-oxidants and antioxidants in the urothelium of 20-month old mice was significantly disturbed. The results thus demonstrate that the intensity of the oxidative stress did not exceed the range of cell survival and that superficial urothelial cells have a strong compensatory mechanism to counteract age-related changes. This protective mechanism against oxidative changes is actually to be expected, since superficial cells are the main component of the blood-urine barrier, which functions as a defence against urine components and reactive species.

### Lipofuscin Accumulation and Ultrastructural Changes of Mitochondria

**Figure 3:**
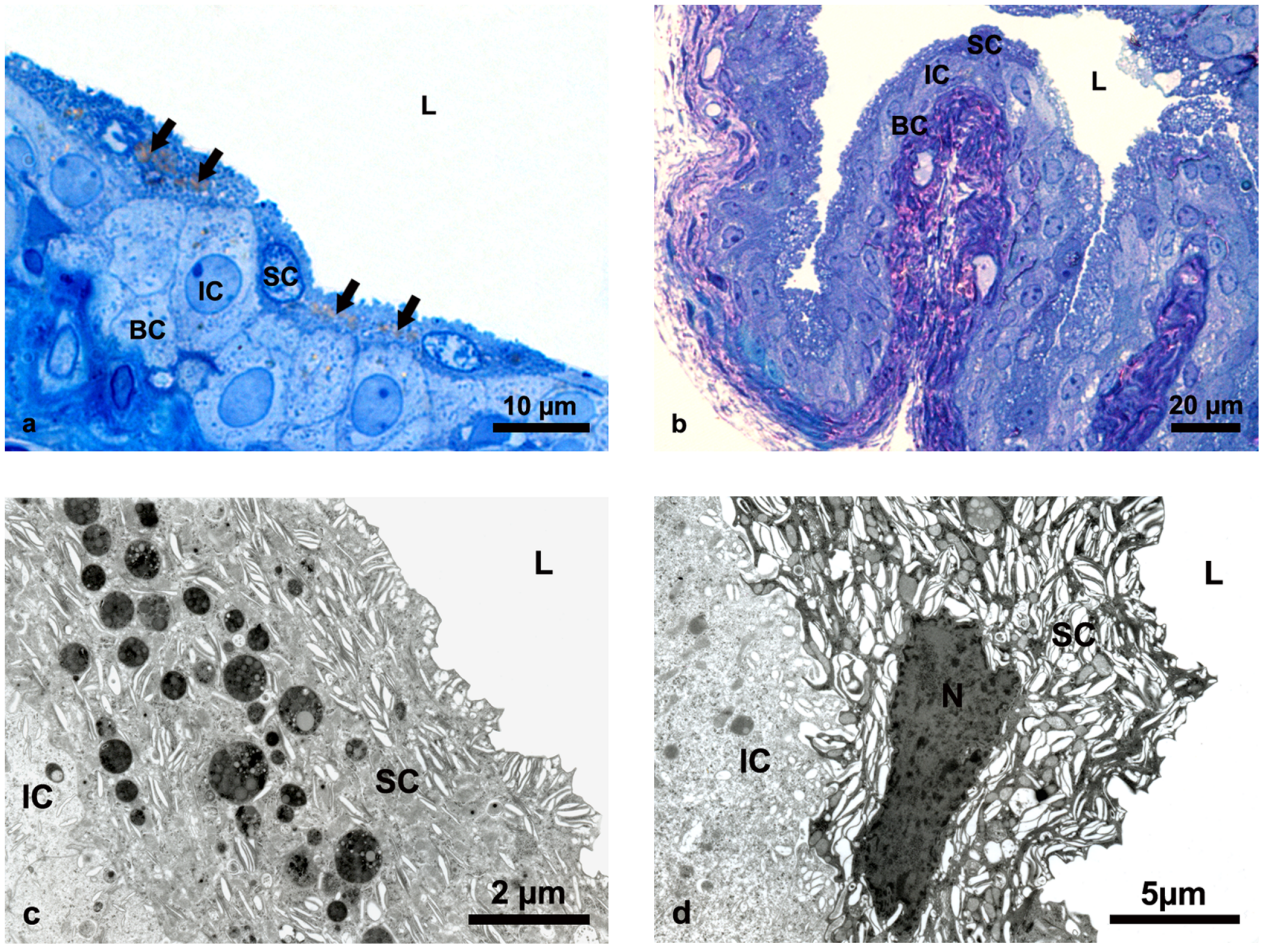
Structure of aging and young mice urothelium. (**a**) Semi-thin section of urothelium in aging mice showing normal structure of three-layered epithelium with numerous lipofuscin granules in the cytoplasm of superficial cells (arrows). (**b**) Semi-thin section of urothelium in young mice showing normal structure of three-layered epithelium with no lipofuscin granules in the superficial cells. (**c**) Ultrathin section of aging urothelium. Ultrastructure of superficial cell fulfiled with specific fusiform vesicles and numerous osmiophilic lipofuscin granules. (**d**) Ultrathin section of young urothelium. Typical ultrastructural appearance of superficial cell with large amounts of fusiform vesicles in the cytoplasm but no lipofuscin granules. SC-superficial cells, IC-intermediate cells, BC-basal cells, N-nucleus, L-lumen of the urinary bladder.

**Figure 4:**
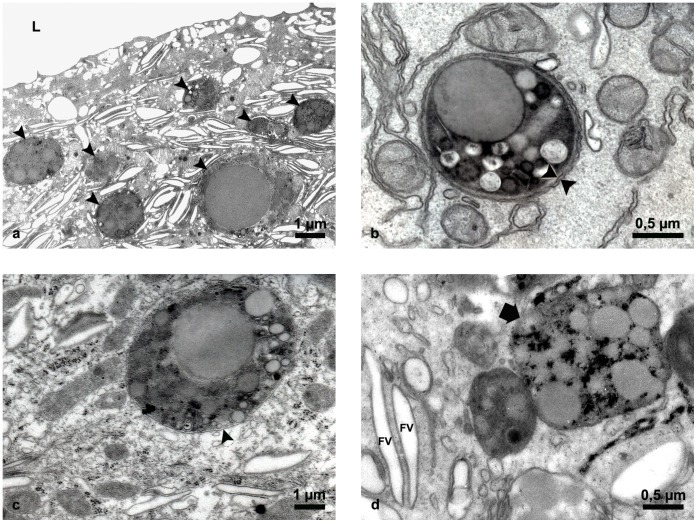
Electron micrographs of lipofuscin in superficial cells of aging mice urothelium. (a) Ultrastructural appearance of lipofuscin granules (arrowheads), heterogeneous in their size and structure. (**b**) Lipofuscin surrounded by double membrane (arrowheads), indicating that the structure is an early autophagic vacuole (autophagosome). (**c**) Lipofuscin in late autophagic vacuole (autophagolysosome), limited by single membrane (arrow). (**d**) The lipofuscin granule (arrow) is filled with electron-dense precipitate as the reaction product of enzyme histochemistry for detection of acid phosphatase activity as the main lysosomal enzyme. Precipitate at the sites of acid phosphatase activity proves that lipofuscin-loaded autophagolysosome contains active lysosomal enzymes trying to degrade the undegradable lipofuscin. FV-fusiform vesicles, typical structures of superficial urothelial cells, L-lumen of the urinary bladder.

**Figure 5:**
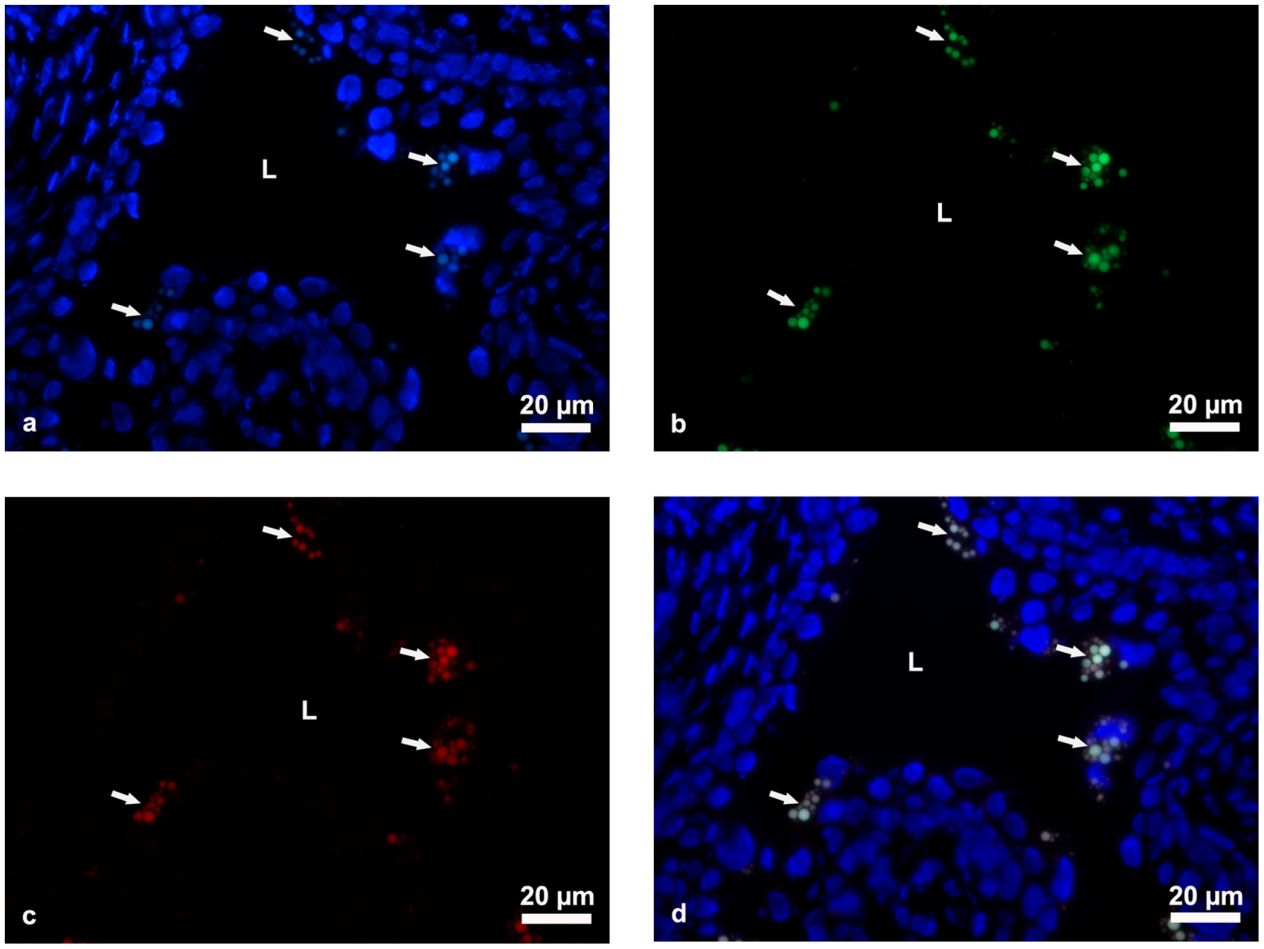
Autofluorescence of lipofuscin granules in superficial cells of aging mice urothelium. Images were taken using ultraviolet (**a**), blue (**b**), green (**c**), ultraviolet and blue (**d**) excitation light. Image (**d**) shows that lipofuscin granules appear exclusively in superficial cells and mostly in the vicinity of their nuclei. Arrows shows clusters of lipofuscin granules. In **a**, **d**: Nuclei are stained with DAPI (blue). L-lumen of the urinary bladder.

However, to investigate structural alterations of cellular organelles we applied various *in situ* methods. Light and transmission electron microscopy showed that the architecture of aging urothelium was normal and the ultrastructure of superficial urothelial cells was unchanged in comparison to young animals (Fig. 3). However, we observed lipofuscin granules in the cytoplasm of superficial urothelial cells in aging urothelium (Fig. 3a, c) but not in the superficial urothelial cells of young mice (Fig. 3b, d). All lipofuscin granules had a regular spherical shape but differed in size and structure, probably due to the varied duration of their maturation process (Fig. 4a). We found lipofuscin in early autophagic vacuoles (autophagosomes), limited by a double membrane (Fig. 4b) and in late autophagic vacuoles (autophagolysosomes), surrounded by a single membrane (Fig. 4c). Since the presence of lipofuscin in the urothelium has not yet been reported, we applied additional reliable methods to examine and confirm our finding. We performed enzyme histochemistry for the detection of acid phosphatase, a widely used method for *in situ* demonstration of lysosomes. We showed that lipofuscin granules are fully lipofuscin-loaded autophagolysosomes (Fig. 4d). We also used fluorescence microscopy additionally to confirm the presence of the autofluorescent age pigment lipofuscin in superficial urothelial cells (Fig. 5).

Lysosomes are known to be one of the cellular organelles in post-mitotic cells that suffer the most remarkable age-related alterations. Lysosomes, which contain enzymes to break down waste materials and cellular debris, progressively accumulate oxidatively modified macromolecules and defective organelles that are not fully degraded. This undegradable, autofluorescent material that accumulates within lysosomes over time is called lipofuscin and is considered to be one of the aging pigments. Lipofuscin has to date been reported in various post-mitotic cells, such as muscle and nerve cells [38]. Our group has for many years investigated the ultrastructure and various cellular processes in urothelial cells of urinary bladder of mice and rats, from their embryonic stage to adulthood (4 months of age) [34], [35], [39]. Throughout the time of our research, we have found no reports or suggestions of the presence of lipofuscin in urothelial cells. To the best of our knowledge, this is the first study showing the presence of lipofuscin in the superficial urothelial cells of urinary bladder. However, in this study, we clearly demonstrated that lipofuscin is present in superficial urothelial cells of 20-month old mice. We also found lipofuscin in the superficial cells of 12-month old mice but to a much smaller extent, indicating that lipofuscin accumulation is an age-dependent process in the urothelium, too.

**Figure 6:**
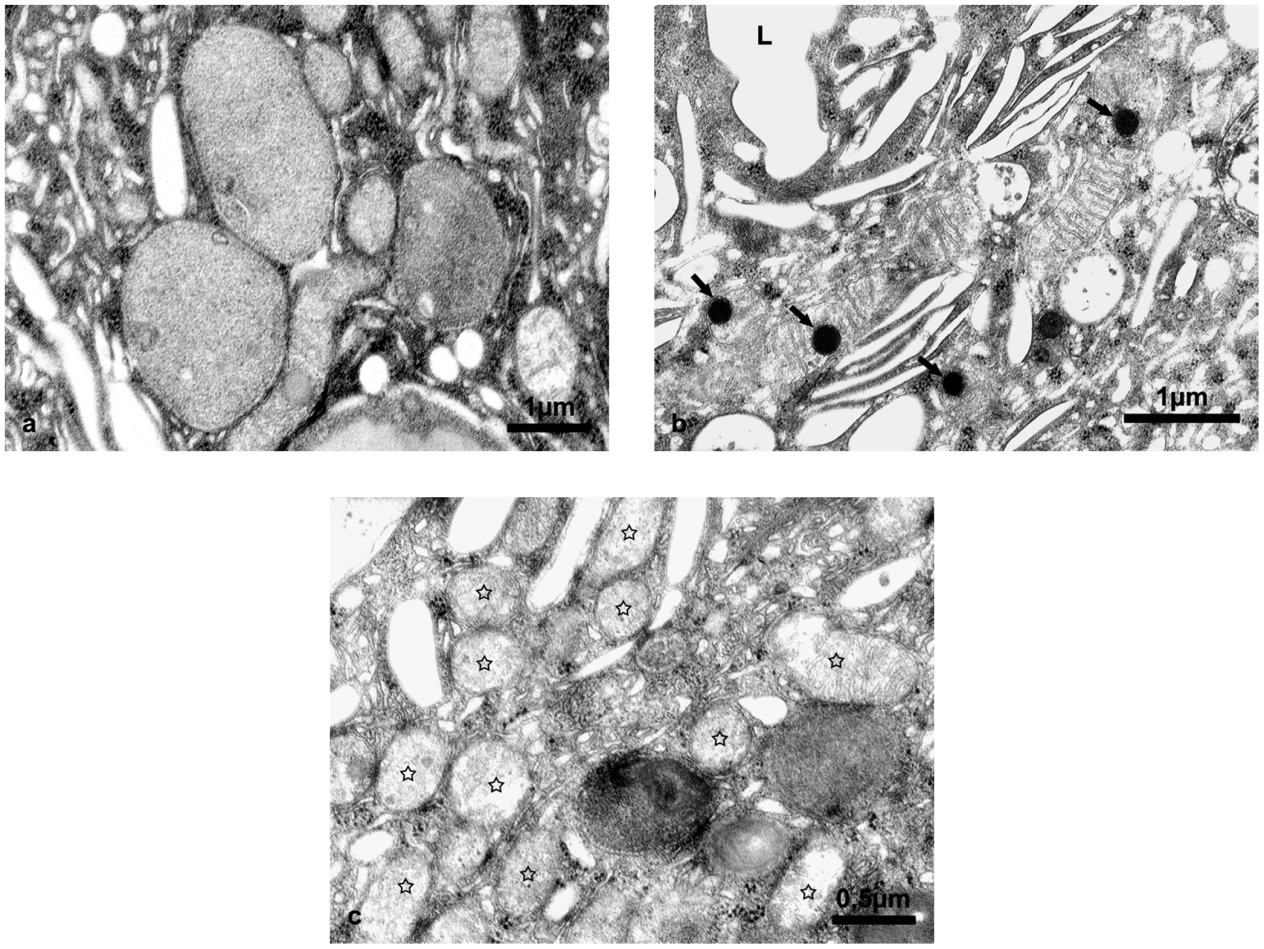
Ultrastructural appearance of mitochondria in superficial cells of aging mice urothelium. (**a**) Enlarged and dilated mitochondria with no visible cristae inside. (**b**) Mitochondria of normal size and shape but with electron-dense inclusions (arrows). (**c**) Mitochondria of normal size and shape but with partial or complete destruction of cristae (star frames). L-lumen of the urinary bladder.

Furthermore, our results also showed that superficial cells of aging mice urothelium had, in addition to normal mitochondria, many swollen mitochondria with partially (just a few visible cristae) or completely destroyed ultrastructure (no visible cristae) (Fig. 6a, c). Numerous mitochondria with electron-dense inclusions were also observed (Fig. 6b). Studies on neurons and myocytes have shown that mitochondria are one of the first cellular components to undergo gradual structural alterations associated with age. These alterations range from partial loss of cristae, swelling to complete destruction of inner membranes and the formation of amorphous electron-dense material [8]. In addition to mitochondria, autophagolysosomes are also known to be one of the cellular organelles in post-mitotic cells that suffer the most remarkable age-related alterations. These organelles, in fact, constantly receive lysosomal hydrolytic enzymes to degrade waste and damaged cellular material. In long-lived post-mitotic cells, autophagolysosomes (autolysosomes) progressively accumulate oxidatively modified proteins and lipids, which form a polymeric and undegradable substance called lipofuscin [28], [40]. Progressive lipofuscin accumulation in autolysosomes results in decreased autophagy. This hampers the autophagic turnover of damaged and senescent mitochondria (which produce a large amount of ROS) and gradually leads to the overload of superficial urothelial cells with damaged mitochondria [27], [40]. All our results demonstrate that a similar process also takes place in the superficial urothelial cells of aging mice.

In conclusion, we showed that lipofuscin accumulation in superficial cells, as well as changes in the activity of antioxidant enzymes, can be found in the urinary bladder of 12-month old mice. Mice at 20 months of age showed decreased antioxidant defence, increased oxidative modification of lipids (demonstrated by increased MDA levels) and proteins (demonstrated by increased iNOS and decreased COX immunoreaction), and structural alterations of cell organelles in the superficial urothelial cells. We clearly showed that significant differences exist in the activity of antioxidants and the antioxidant status in bladder urothelial cells between young (2 months) and aging (20 months) mice. In spite of the markedly elevated activity of the main antioxidants (CAT, GPx, GR, GSH), total antioxidant capacity in the urothelium of aging mice was decreased and numerous oxidative alterations were induced (increased MDA and iNOS and decreased COX). We also found ultrastructural alterations in mitochondria and large lipofuscin deposits in the superficial cells of the urinary bladder of aging mice. All these results undoubtedly demonstrate that superficial urothelial cells of aging mice possess changes in oxidative status and structural alterations similar to those of other long-lived post-mitotic cells. This remarkable finding suggests that progressive accumulation of age-related structural alterations in urothelial cells may contribute to various age-related urinary diseases. However, this assumption is based on logic and needs to be examined further. Investigation of the molecular mechanisms involved in the aging process of urothelial cells is currently being carried out in our laboratory.

## Materials and Methods

### Animals

Fifteen young (adult, sexually mature 2 months old) and 15 aging (20 months old) female C57BL/6JOlaHsd mice (Medical Experimental Centre, Ljubljana, Slovenia) were used for this study, in accordance with European guidelines and Slovenian legislation. The mice were housed 5 per cage (825 cm^2^ floor space) on Lignocel ¾ bedding material (Germany) at a 22–23°C room temperature, 55±10% humidity and a 12 h light/dark cycle (illumination between 07.00 p.m. - 07.00 a.m.) and received food (Altromin 1314, Germany) and tap water *ad libitum*. The mice were sacrificed by CO_2_ inhalation at the beginning of the light phase of the day between 8 and 10 a.m., and their blood and urinary bladders were taken immediately after sacrifice. The experiment was approved by the Veterinary Administration of the Republic of Slovenia (license number 34401-5/2009/4) and was conducted in accordance with the European Convention for the protection of vertebrate animals used for experimental and other scientific purposes (ETS 123) as well as in accordance with National Institutes of Health guidelines for work with laboratory animals.

### Antioxidant Status Determination

Following euthanasia, blood was taken by heart puncture after opening the thoracic region and was allowed to clot for approximately 2 h at room temperature. Serum was prepared by centrifugation (200 g, 5 min, RT) and stored at −80°C until used. The urinary bladder was removed and cut into halves. The urothelium was peeled off from the bladder wall of each half, placed in tubes, snap frozen in liquid nitrogen and stored at −80°C for later analysis.

Urinary bladder epithelium and serum were then homogenized in Tris-buffer solution (pH 7.4; organ: buffer 1∶10; w/w) and divided into two portions. One was used for MDA (Chromsystems Diagnostic, Munchen, Germany) determination and the second was centrifuged at 13,000×g for 20 min at 4°C (Beckman refrigerated, Ultracentrifuge).

The supernatant was used to assay total protein concentration (TP), GSH, GPx, GR, SOD, CAT and total antioxidant status (TAS). Free GSH and GSSG levels were measured with a Chromsystems Diagnostic commercial kit for HPLC analysis (Munchen, Germany) and were expressed as mg/L. The ratio of free GSH/GSSG was also calculated. An HPLC Agilent HP 1100-model system equipped with an autosampler and a fluorescence detector (Waldbronn, Germany) was used. The Randox commercial systems, Ransel (GPx), Ransod (SOD), total antioxidant status (TAS) and GR (Crumlin, United Kingdom) and Sentinel Diagnostics (Milan, Italy) were used to determine the level of GPx, SOD, TAS, GR and TP, respectively. CAT activity in the homogenate was quantified according to the method by Aebi [41], in which hydrogen peroxide was reacted with the cell lysates. The initial rate of hydrogen peroxide disappearance (0–60 s) was recorded spectrophotometrically at a wavelength of 240 nm. The results were expressed as U/mg TP (SOD, CAT), U/g TP (GPx, GR) and mM (TAS).

TAS was measured with a kit, wherein 2,2′-azinobis-(3-ethylbenzthiazoline sulphonate) (ABTS) was incubated with a peroxidase (metmyoglobin) and hydrogen peroxide to produce the radical cation ABTS^·+^, which has a relatively stable blue-green colour and can be measured at 600 nm. Antioxidants in the sample caused suppression of the production of this colour to a degree that was proportional to their concentration.

### Immunostaining of Paraffin Sections

The urinary bladders were fixed in 4% paraformaldehyde overnight and embedded in paraffin. After deparaffinization through graded alcohol, 5 µm thick specimens were microwave heated for 5 min at 600 W for antigen retrieval. Endogenous peroxidase activity was blocked by incubation in 3% H_2_O_2_ in methanol for 15 min. After washing in phosphate-buffered saline (PBS), sections were blocked in normal swine serum (1∶10; Sigma, Taufkirchen, Germany) for 1 h at room temperature and incubated overnight at 4°C with polyclonal rabbit antibodies against iNOS (1∶100; Abcam, Cambridge, UK). As negative controls, incubation in primary antibodies was replaced with incubation in serum from non-immunized animals. After washing in PBS, biotinylated swine anti-rabbit antibodies (1∶200) were applied for 1 h at 37°C, followed by incubation with ABC complex/HRP (Dako, Glostrup, Denmark) for 30 min at room temperature. After the standard DAB (Sigma, Taufkirchen, Germany) development procedure, sections were stained in Mayer’s haematoxylin, dehydrated, mounted in Neo-Mount (Merck, Darmstadt, Germany) and examined with a Nikon Eclipse TE bright-field microscope (Amstelveen, Netherlands).

### Immunofluorescent Labelling of Cryosections

Excised urinary bladders were fixed for 2 h using 3% paraformaldehyde in phosphate-buffered saline (PBS), rinsed overnight in 30% sucrose, embedded in Jung tissue freezing medium (Leica Microsystems Nussloch GmbH, Nussloch, Germany), frozen and cut into 7 µm thick cryosections with a cryostat (CM3000, Leica, Germany). After air-drying for at least 3 h and washing in PBS, sections were permeabilized in cold methanol for 5 min, washed in PBS, blocked in 5% foetal calf serum (FCS) and 1% bovine serum albumin (BSA) in PBS for 2 h at 37°C and incubated overnight at 4°C with appropriate primary antibodies as follows: mouse monoclonal against COX (Abcam, Cambridge, UK), diluted 1∶500; rat monoclonal against Ki-67 (clone MIB-1, Dako, Glostrup, Denmark), diluted 1∶100; rabbit polyclonal against active caspase 3 (Abcam, Cambridge, UK), diluted 1∶300; rabbit polyclonal anti-uroplakin (kind gift from Prof. T.T. Sun), diluted 1.10000; and mouse monoclonal against CK20 (Dako, Glostrup, Denmark), diluted 1∶100. After washing in PBS, sections were incubated with suitable secondary antibodies as follows: tetramethyl rhodamine iso-thiocyanate (TRITC)-labelled goat anti-rabbit or goat anti-rat (Alexa Fluor® 555; Invitrogen, Molecular Probes, Leiden, Netherlands), diluted 1∶200 for 1 h at 37°C in the dark or fluorescein iso-thiocyanate (FITC)-labelled goat anti-mouse (Alexa Fluor® 488; Invitrogen, Molecular Probes, Leiden, Netherlands), diluted 1∶200 for 1 h at 37°C in the dark. After washing in PBS, the samples were mounted in Vectashield antibleaching mounting medium with 4′,6-diamidino-2-phenylindole (DAPI) (Vector Laboratories, Burlingame, CA, USA) for staining DNA. Appropriate negative controls, in which the primary antibodies were replaced with serum from non-immunized animals, were performed. The samples were analyzed with a Nikon Eclipse TE fluorescence microscope (Amstelveen, Netherlands).

### Transmission Electron Microscopy

Excised urinary bladders were cut into small pieces and fixed for 3 h at 4°C in a mixture of 4% paraformaldehyde and 2% glutaraldehyde in 0.2 M cacodylate buffer (pH 7.3). Overnight rinsing in 0.33 M sucrose in 0.2 M cacodylate buffer was followed by post-fixation with 1% OsO_4_ for 1 h. After dehydration in ethanol series, tissue samples were embedded in Epon (Serva Electrophoresis, Heidelberg, Germany). Ultrathin sections were contrasted with uranyl acetate and lead citrate and examined with a Jeol 100 CX electron microscope.

Epon semi-thin sections (1 µm) were stained with 1% Toluidine blue and 2% borate in distilled water for 20 seconds and observed with a Nikon Eclipse TE bright field microscope.

### Enzyme Histochemistry for the Detection of Acid Phosphatase Activity

Excised urinary bladders were cut into small pieces and fixed for 1 h at 4°C using 2% glutaraldehyde and 5% sucrose in 0.1 M cacodylate buffer (pH 7.4). Overnight rinsing at 4°C with 0.33 M sucrose in 0.1 M cacodylate buffer was followed by incubation for 40 min at 37°C in a solution of 0.1 M sodium β-glycerophosphate and 0.002 M Pb(NO_3_)_2_ in 0.2 M tris maleate buffer (pH 5.0). After rinsing with 0.2 M tris maleate buffer, tissue pieces were postfixed for 1 h at 4°C in a mixture of 2% OsO_4_ and 3% potassium ferrocyanide in 0.2 M cacodylate buffer. After dehydration in ethanol series, tissue pieces were embedded in Epon (Serva Electrophoresis, Heildelberg, Germany). Ultrathin sections were contrasted with lead citrate and examined with a Jeol 100 CX electron microscope.

### Detection of Autofluorescence of Lipofuscin on Cyro-sections

Excised urinary bladders were cut into halves, fixed for 2 h using 3% paraformaldehyde in phosphate-buffered saline (PBS), rinsed overnight in 30% sucrose, embedded in Jung tissue freezing medium (Leica Microsystems Nussloch GmbH, Nussloch, Germany), frozen and cut into 7µm thick cryosections with a cryostat (CM3000, Leica, Germany). After air-drying for at least 3 h and washing in PBS, specimens were mounted in Vectashield antibleaching mounting medium with 4′,6-diamidino-2-phenylindole (DAPI) (Vector Laboratories, Burlingame, CA, USA) for staining DNA, and observed under ultraviolet (excitation filter: 330–380 nm), blue (excitation filter: 450–490 nm) and green (excitation filter: 510–560 nm) excitation light with a Nikon Eclipse TE fluorescence microscope (Amstelveen, Netherlands).

### Statistical Analysis

Differences in enzymic and non-enzymic antioxidants were analyzed by comparison of two samples. Significant differences between groups of young and aging mice were evaluated by a test of independence (t test) and when variances of the two samples were different, we used the Mann-Whitney (Wilcoxon) W test. Age-related differences in the levels of antioxidants and LDH in the urothelium of 2, 12 and 20 months old mice were analyzed by Multivariate Analysis (MANOVA) and significant differences among groups were evaluated by multiple range tests using Duncun method. The results were expressed as the mean ± SEM and were considered statistically significant at P<0.05. All statistical analyses were made using the Statgraphics® Centurion XV computer program.

## Supporting Information

Figure S1 Age-related differences in the levels of antioxidants and malondialdehyde in the urothelium (uroth.) and serum of young (8 week) and aging (80 weeks) C57BL/6JOlaHsd female mice. CAT, catalase; GPx, glutathione peroxidase; GR, glutathione reductase; GSH, glutathione; MDA, malondialdehyde; SOD, superoxide dismutase.
(DOCX)

Figure S2 Age-related differences in the level of total antioxidant status (TAS) in the urothelium of young (8 week) and aging (80 weeks) C57BL/6JOlaHsd female mice.
(DOCX)

Table S1 Age-related differences in the levels of antioxidants and lactate dehydrogenase in the urothelium of 2, 12 and 20 months old C57BL/6JOlaHsd female mice. Data were analyzed by Multivariate Analysis (MANOVA) and significant differences among groups were evaluated by multiple range tests using Duncun method. The results were considered statistically significant at P<0.05. Legend: ns, non-significant; CAT, catalase; GPx, glutathione peroxidase; GR, glutathione reductase; LDH, lactate dehydrogenase; SOD, superoxide dismutase; TP, total protein concentration. Values are mean ± SEM. Values with different superscript letters in the same row are significantly different.
(DOCX)
