# Supplementary material for: Oxidative Status and Lipofuscin Accumulation in Urothelial Cells of Bladder in Aging Mice
Source: PLoS One. 2013 Mar 20;8(3):e59638. doi: 10.1371/journal.pone.0059638 (PMC3603863; doi:10.1371/journal.pone.0059638)
Supplement: Table S1 — Age-related differences in the levels of antioxidants and lactate dehydrogenase in the urothelium of 2, 12 and 20 months old C57BL/6JOlaHsd female mice. Data were analyzed by Multivariate Analysis (MANOVA) and significant differences among groups were evaluated by multiple range tests using Duncun method. The results were considered statistically significant at P<0.05. Legend: ns, non-significant; CAT, catalase; GPx, glutathione peroxidase; GR, glutathione reductase; LDH, lactate dehydrogenase; SOD, superoxide dismutase; TP, total protein concentration. Values are mean ± SEM. Values with different superscript letters in the same row are significantly different. (DOCX) [file pone.0059638.s003.docx]

| **Parameter** | **young (2 months)** | **(12 months)** | **aging (20 months)** | **P value** |
| --- | --- | --- | --- | --- |
| **TP (g/L)** | 6.2±1.2 | 6.1±1.7 | 8.6±0.9 | ns |
| **SOD (U/mg TP)** | 7.13±1.32 | 8.0±3.4 | 8.15±1.38 | ns |
| **CAT (U/mg TP)** | 0.16±0.01^a^ | 0.71±0.24^ab^ | 1.08±0.14^b^ | < 0.001 |
| **GR (U/g TP)** | 1.64±0.35^a^ | 11.5±3.7^b^ | 5.15±0.32^a^ | < 0.001 |
| **GPx (U/g TP)** | 10.8±1.9^a^ | 16.5±6.9^ab^ | 29.0±2.8^b^ | < 0.001 |
| **LDH (U/L)** | 381.3±51.4^a^ | 1361.0±123.5^b^ | 1009.3±110.1^b^ | < 0.001 |

Values are mean ± SEM. Values with different superscript letter in the same row are significantly different.

Legend: ns, non-significant; CAT, catalase; GPx, glutathione peroxidase; GR, glutathione reductase; LDH – lactate dehydrogenase SOD, superoxide dismutase; TP, total protein concentration.
