# Supplementary material for: Oxidative Status and Lipofuscin Accumulation in Urothelial Cells of Bladder in Aging Mice
Source: PLoS One. 2013 Mar 20;8(3):e59638. doi: 10.1371/journal.pone.0059638 (PMC3603863; doi:10.1371/journal.pone.0059638)
Supplement: Supplementary file 2 [file pone.0059638.s001.docx]

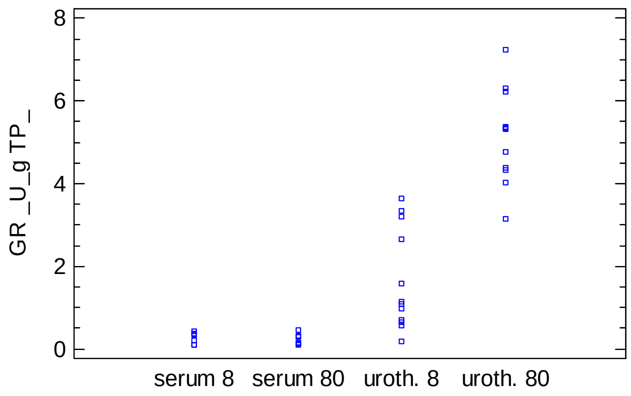
GR _U_g TP_
0
2
4
6
8
serum 8
serum 80
uroth. 8
uroth. 80

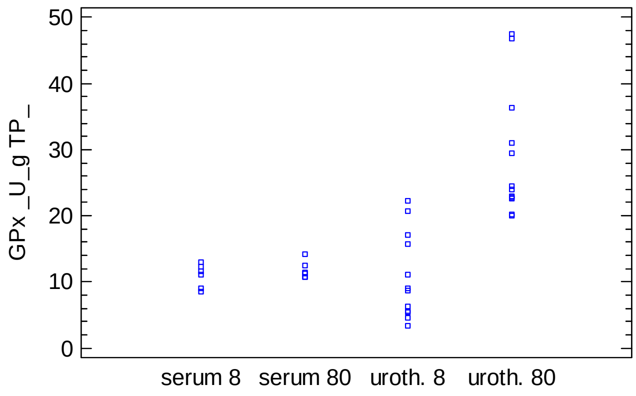
GPx _U_g TP_
0
10
20
30
40
50
serum 8
serum 80
uroth. 8
uroth. 80

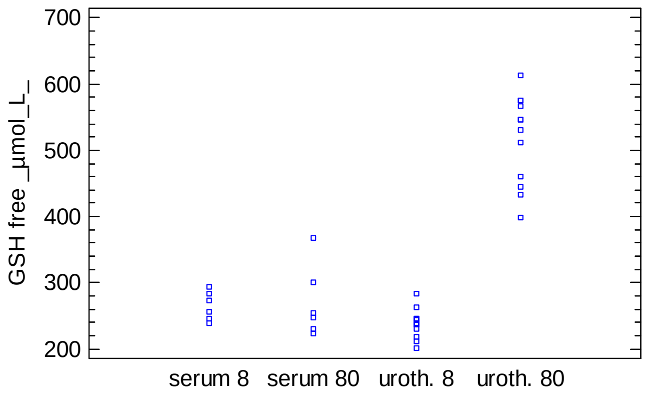
GSH free _µmol_L_
200
300
400
500
600
700
serum 8
serum 80
uroth. 8
uroth. 80

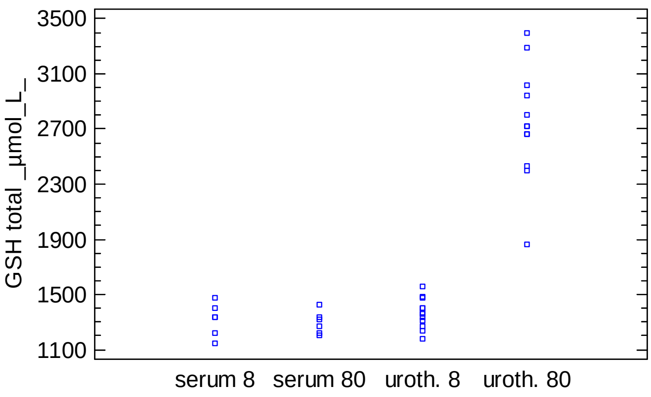
GSH total _µmol_L_
1100
1500
1900
2300
2700
3100
3500
serum 8
serum 80
uroth. 8
uroth. 80

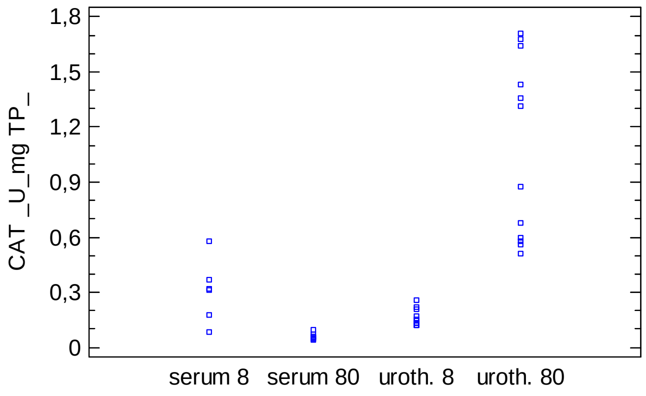
CAT _U_mg TP_
0
0,3
0,6
0,9
1,2
1,5
1,8
serum 8
serum 80
uroth. 8
uroth. 80

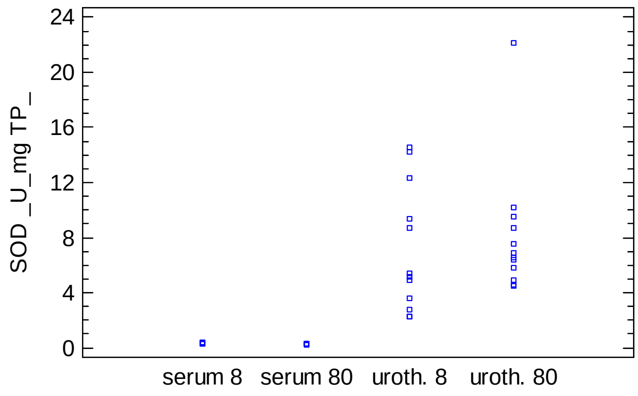
SOD _U_mg TP_
0
4
8
12
16
20
24
serum 8
serum 80
uroth. 8
uroth. 80

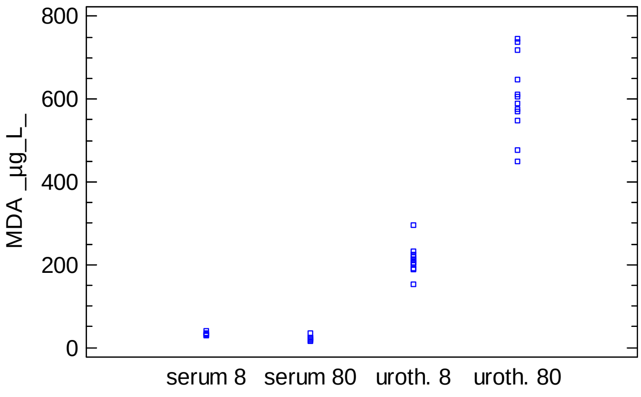
MDA _µg_L_
0
200
400
600
800
serum 8
serum 80
uroth. 8
uroth. 80
