# Supplementary material for: Oxidative Status and Lipofuscin Accumulation in Urothelial Cells of Bladder in Aging Mice
Source: PLoS One. 2013 Mar 20;8(3):e59638. doi: 10.1371/journal.pone.0059638 (PMC3603863; doi:10.1371/journal.pone.0059638)
Supplement: Supplementary file 3 [file pone.0059638.s002.docx]

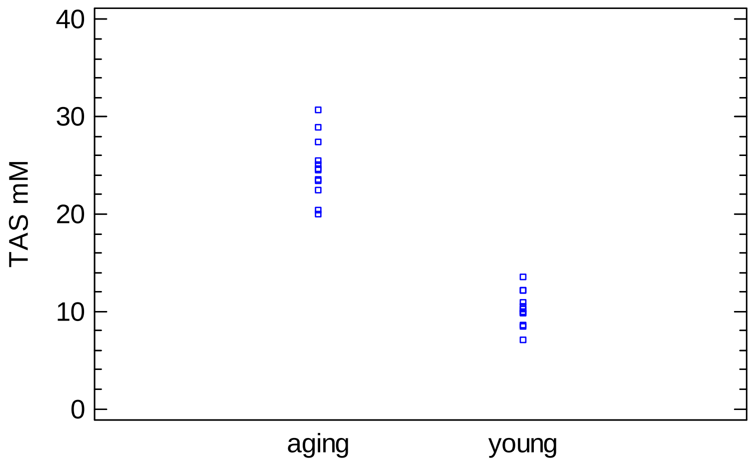

TAS mM
40
30
20
10
0
aging
young
